# Unraveling plant hormone signaling through the use of small molecules

**DOI:** 10.3389/fpls.2014.00373

**Published:** 2014-07-30

**Authors:** Adeline Rigal, Qian Ma, Stéphanie Robert

**Affiliations:** Department of Forest Genetics and Plant Physiology, Umeå Plant Science Centre, Swedish University of Agricultural SciencesUmeå, Sweden

**Keywords:** phytohormones, hormone signaling, structure-activity relationship, labeled molecule, agonists and antagonists

## Abstract

Plants have acquired the capacity to grow continuously and adjust their morphology in response to endogenous and external signals, leading to a high architectural plasticity. The dynamic and differential distribution of phytohormones is an essential factor in these developmental changes. Phytohormone perception is a fast but complex process modulating specific developmental reprogramming. In recent years, chemical genomics or the use of small molecules to modulate target protein function has emerged as a powerful strategy to study complex biological processes in plants such as hormone signaling. Small molecules can be applied in a conditional, dose-dependent and reversible manner, with the advantage of circumventing the limitations of lethality and functional redundancy inherent to traditional mutant screens. High-throughput screening of diverse chemical libraries has led to the identification of bioactive molecules able to induce plant hormone-related phenotypes. Characterization of the cognate targets and pathways of those molecules has allowed the identification of novel regulatory components, providing new insights into the molecular mechanisms of plant hormone signaling. An extensive structure-activity relationship (SAR) analysis of the natural phytohormones, their designed synthetic analogs and newly identified bioactive molecules has led to the determination of the structural requirements essential for their bioactivity. In this review, we will summarize the so far identified small molecules and their structural variants targeting specific phytohormone signaling pathways. We will highlight how the SAR analyses have enabled better interrogation of the molecular mechanisms of phytohormone responses. Finally, we will discuss how labeled/tagged hormone analogs can be exploited, as compelling tools to better understand hormone signaling and transport mechanisms.

## Introduction

Plants produce a wide variety of endogenous small molecules, allowing them to thrive in the face of internal or external challenges. Among these molecules, phytohormones are growth regulators, which are effective at low concentrations, controlling a vast range of developmental and adaptive processes (Rubio et al., 2009). Our comprehension of plant hormone biology (metabolism, transport, perception, and signaling) has increased tremendously during the last decade. Most of this knowledge has been gained using genetic approaches in the model plant *Arabidopsis thaliana*, however in recent years, chemical genetics has been introduced as a compelling tool in plant science. The application of small molecules allows instantaneous, reversible and conditional alteration of a phenotype and thereby offers circumvention of the limitations of classical genetic approaches, including genetic redundancy, lethality and pleiotropism (Toth and Van Der Hoorn, 2010). Chemical genetics has been extensively employed to study molecular mechanisms of complex and highly dynamic processes such as plant hormone signaling, leading to new possibilities and perspectives in hormone biology. This new knowledge on plant hormone chemistry has not only led to the identification of structurally related compounds for commercial applications, but has also and most importantly provided the basis for the rational design of novel analog molecules as chemical tools probing phytohormone-regulated responses. Determination of the bioactive moieties of many phytohormone molecules in combination with synthetic chemistry has generated an assortment of novel compounds including phytohormone agonists and antagonists and tagged/labeled phytohormone-analogous molecules. Application of those compounds has contributed significantly to our current understanding of the modes of action of phytohormones. Thus, the inter-connection between chemistry and plant biology provides new insights into plant hormone biology. Here, we will review some prominent examples of the use of chemical genomic strategies in plant hormone research. We will focus on abscisic acid (ABA), salicylic acid (SA), auxin, cytokinin (CK), brassinosteroid (BR), and strigolactone (SL) signaling pathways. The review by Chini and co-authors in this issue covers similar topic for jasmonate related-research. This review will highlight how the integration between chemistry and biology improves the potential to dissect hormone signaling.

## Agonist and antagonist molecules

### ABA agonists and antagonists

**Figure 1:**
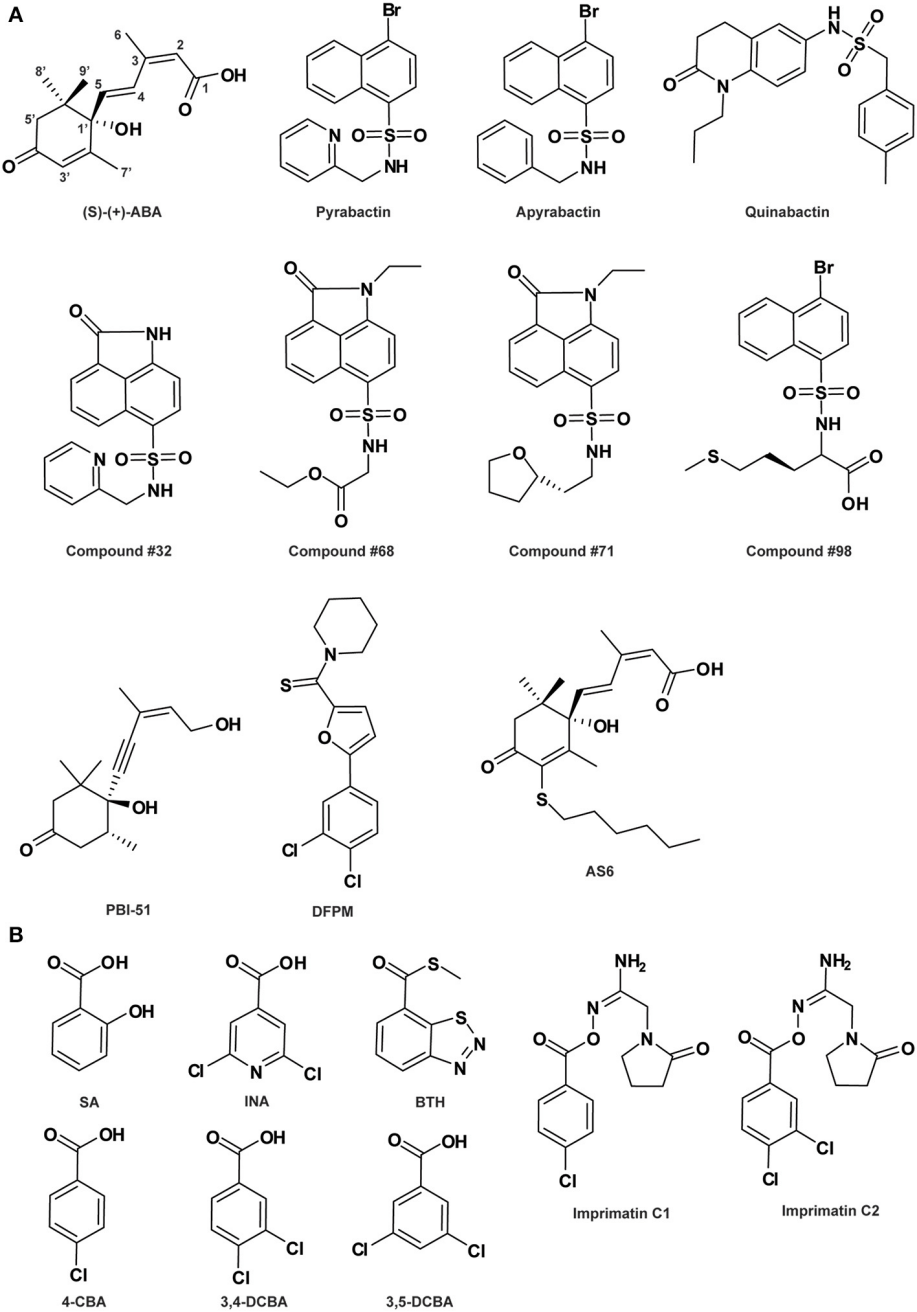
**Abscisic acid- (A) and salicylic acid-related compounds (B)**. See Table 1 for the full name of each compound.

ABA (Figure 1A) is a sesquiterpenoid plant hormone, which is involved in both biotic/abiotic stress responses and regulation of important aspects of plant growth and development (Cutler et al., 2010). Based on a chemical biology strategy, a variety of small ABA-related bioactive compounds have been identified or designed with the aim to elucidate the mode of action of ABA in plants (Kitahata and Asami, 2011). The most salient example is the selective ABA agonist named pyrabactin (Figure 1A), which inhibits seed germination but has no effect on other ABA responses (Zhao et al., 2007; Park et al., 2009). Genetic isolation of mutants insensitive to pyrabactin in a seed germination assay led to the identification of PYRABACTIN RESISTANCE 1 (PYR1) as well as 13 PYR1-like (PYL) members, a new class of START domain proteins, as the long-sought-after intracellular ABA receptors in *Arabidopsis* (Park et al., 2009). Structural biology analyses using ABA/pyrabactin-bound receptors revealed a gate-latch-lock mechanism for ABA perception (Melcher et al., 2009; Miyazono et al., 2009; Nishimura et al., 2009; Santiago et al., 2009; Yin et al., 2009): ligand binding causes conformational changes in these receptor proteins, which induces closure of the “gate” and “latch” loops surrounding the ligand-binding pocket. Ligand-induced closure of the gate creates an interaction surface required for binding TYPE 2C PROTEIN PHOSPHATASES (PP2Cs), which are negative regulators of ABA signaling (Merlot et al., 2001; Leonhardt et al., 2004; Saez et al., 2004; Yoshida et al., 2006; Nishimura et al., 2007). With no or low concentration of ABA, PP2Cs like ABA INSENSITIVE 1 (ABI1), ABI2, HOMOLOGY TO ABI1 (HAB1) and PP2CA/ ABA-HYPERSENSITIVE GERMINATION 3 (AHG3), suppress ABA responses by dephosphorylating and inactivating downstream SUCROSE NON-FERMENTING-1 (SNF1)-RELATED PROTEIN KINASE 2 (SnRK2) kinases, the positive regulators in ABA signaling (Gómez-Cadenas et al., 1999; Mustilli et al., 2002; Fujii et al., 2007; Fujii and Zhu, 2009; Nakashima et al., 2009). An increase in ABA level inhibits the phosphatase activity of PP2C via the formation of an ABA-receptor-PP2C ternary complex, thereby allowing SnRK2s to be activated by phosphorylation (Cutler et al., 2010; Weiner et al., 2010; Miyakawa et al., 2013). Activated SnRK2s in turn phosphorylate and activate downstream effectors mediating various ABA responses (Kobayashi et al., 2005; Furihata et al., 2006; Cutler et al., 2010). The selectivity of pyrabactin for a subset of the PYR/PYL ABA receptors has been exploited to effectively bypass the genetic redundancy in the *pyr*/*pyl* gene family, which was always eluded in classical genetic mutation analyses (Park et al., 2009).

**Table 1:** **Names of the phytohormone-related chemical compounds described in the review**.

**Common name**	**IUPAC name**
Abscisic acid (ABA)	(2Z,4E)-5-((S)-1-hydroxy-2,6,6-trimethyl-4-oxocyclohex-2-enyl)-3-methylpenta-2,4-dienoic acid
Pyrabactin	4-bromo-N-(pyridin-2-ylmethyl)naphthalene-1-sulfonamide
Apyrabactin	N-benzyl-4-bromonaphthalene-1-sulfonamide
Quinabactin	N-(2-oxo-1-propyl-1,2,3,4-tetrahydroquinolin-6-yl)-1-p-tolylmethanesulfonamide
Compound #32	2-oxo-N-(pyridin-2-ylmethyl)-1,2-dihydrobenzo[cd]indole-6-sulfonamide
Compound #68	ethyl 2-(1-ethyl-2-oxo-1,2-dihydrobenzo[cd]indole-6-sulfonamido)acetate
Compound #71	(S)-1-ethyl-2-oxo-N-(2-(tetrahydrofuran-2-yl)ethyl)-1,2-dihydrobenzo[cd]indole-6-sulfonamide
Compound #98	2-(4-bromonaphthalene-1-sulfonamido)-5-(methylthio)pentanoic acid
PBI-51	(4S,5R)-4-hydroxy-4-((Z)-5-hydroxy-3-methylpent-3-en-1-ynyl)-3,3,5-trimethylcyclohexanone
DFPM	(5-(3,4-dichlorophenyl)furan-2-yl)(piperidin-1-yl)methanethione
3′-hexylsulfanyl-ABA (AS6)	(2Z,4E)-5-((S)-3-(hexylthio)-1-hydroxy-2,6,6-trimethyl-4-oxocyclohex-2-enyl)-3-methylpenta-2,4-dienoic acid
Salicylic acid (SA)	2-hydroxybenzoic acid
INA	2,6-dichloroisonicotinic acid
Benzothiadiazole (BTH)	S-methyl 1,2,3-benzothiadiazole-7-carbothioate
Imprimatin C1	(E)-N′-(4-chlorobenzoyloxy)-2-(2-oxopyrrolidin-1-yl)acetimidamide
Imprimatin C2	(E)-N′-(3,4-dichlorobenzoyloxy)-2-(2-oxopyrrolidin-1-yl)acetimidamide
4-CBA	4-chlorobenzoic acid
3,4-DCBA	3,4-dichlorobenzoic acid
3,5-DCBA	3,5-dichlorobenzoic acid
IAA	indol-3-acetic acid
NAA	1-naphthaleneacetic acid
2,4-D	2,4-dichlorophenoxyacetic acid
Picloram	4-amino-3,5,6-trichloro-2-pyridinecarboxylic acid
5-F-IAA	5-fluoro-indol-3-acetic acid
BH-IAA	8-(tert-butoxycarbonylamino)-2-(1H-indol-3-yl)octanoic acid
PEO-IAA	2-(1H-indol-3-yl)-4-oxo-4-phenylbutanoic acid
Auxinole	4-(2,4-dimethylphenyl)-2-(1H-indol-3-yl)-4-oxobutanoic acid
FITC-IAA	2-(1-(3′,6′-dihydroxy-3-oxo-3H-spiro[isobenzofuran-1,9′-xanthene]-5-ylcarbamothioyl)-1H-indol-3-yl)acetic acid
RITC-IAA	N-(9-(2-carboxy-6-(3-(carboxymethyl)-1H-indole-1-carbothioamido)phenyl)-6-(diethylamino)-3H-xanthen-3-ylidene)-N-ethylethanaminium
Terfestatin A (TrfA)	(2S,3R,4S,5S,6R)-2-(2,4-dihydroxy-3,6-diphenylphenoxy)-6-(hydroxymethyl)oxane-3,4,5-triol
trans-Zeatin (tZ)	(E)-4-(9H-purin-6-ylamino)-2-methylbut-2-en-1-ol
N^6^-(2-hydroxy-3-methylbenzylamino) purine (PI-55)	2-((9H-purin-6-ylamino)methyl)-6-methylphenol
N^6^-(2,5-dihydroxybenzylamino) purine (LGR-991)	2-((9H-purin-6-ylamino)methyl)benzene-1,4-diol
N^6^-(benzyloxymethyl) adenosine (BOMA)	(2R,3R,4S,5R)-2-(6-(benzyloxymethylamino)-9H-purin-9-yl)-5-(hydroxymethyl)tetrahydrofuran-3,4-diol
S-4893	3-(6-chloro-4-phenylquinazolin-2-ylamino)propan-1-ol
N^6^-benzyladenine (BA)/6-benzylaminopurine (BAP)	N-benzyl-7H-purin-6-amine
Brassinolide (BL)	(3aS,5S,6R,7aR,9aS,10R)-10-((2S,3S,4S,5R)-3,4-dihydroxy-5,6-dimethylheptan-2-yl)-5,6-dihydroxy-7a,9a-dimethyltetradecahydro-1H-benzo[c]indeno[5,4-e]oxepin-3(12bH)-one
Bikinin (BIK)	4-(5-bromopyridin-2-ylamino)-4-oxobutanoic acid
Brassinopride (BRP)	N-benzyl-N-(1-cyclopropylethyl)-4-fluorobenzamide
Castasterone (CS)	(2R,3S,5S,10R,13S)-17-((2S,3S,4S,5S)-3,4-dihydroxy-5,6-dimethylheptan-2-yl)-2,3-dihydroxy-10,13-dimethyltetradecahydro-1H-cyclopenta[a]phenanthren-6(10H)-one
Alexa Fluor 647-castasterone (AFCS)	2-((1E,3E,5Z)-5-(3-(6-(5-(2-((E)-((2R,3S,10R,13S)-17-((2S,3S,4S,5S)-3,4-dihydroxy-5,6-dimethylheptan-2-yl)-2,3-dihydroxy-10,13-dimethyloctahydro-1H-cyclopenta[a]phenanthren-6(10H,12H,13H,14H,15H,16H,17H)-ylidene)aminooxy)acetamido)pentylamino)-6-oxohexyl)-3-methyl-5-sulfo-1-(3-sulfopropyl)indolin-2-ylidene)penta-1,3-dienyl)-3,3-dimethyl-5-sulfo-1-(3-sulfopropyl)-3H-indolium
(+)-Strigol	(3aR,5S,8bS,E)-5-hydroxy-8,8-dimethyl-3-(((R)-4-methyl-5-oxo-2,5-dihydrofuran-2-yloxy)methylene)-3,3a,4,5,6,7,8,8b-octahydro-2H-indeno[1,2-b]furan-2-one
Karrikin1 (KAR1)	3-methyl-2H-furo[2,3-c]pyran-2-one
GR24	(3aR,8bS,E)-3-(((R)-4-methyl-5-oxo-2,5-dihydrofuran-2-yloxy)methylene)-3,3a,4,8b-tetrahydro-2H-indeno[1,2-b]furan-2-one
Cyano-isoindole-strigolactone-analog-1 (CISA-1)	(E)-ethyl 2-(1-(but-3-enyl)-3-cyano-2H-isoindol-2-yl)-3-(4-methyl-5-oxo-2,5-dihydrofuran-2-yloxy)acrylate
4-Br debranone	5-(4-bromophenoxy)-3-methylfuran-2(5H)-one
3′-methyl-GR24	(3aR,8bS,Z)-3-(((R)-3,4-dimethyl-5-oxo-2,5-dihydrofuran-2-yloxy)methylene)-3,3a,4,8b-tetrahydro-2H-indeno[1,2-b]furan-2-one
tia-3′-methyl-debranones-like molecule	5-((4-Chlorophenyl)thio)-3,4-dimethylfuran-2(5H)-one
AR36	(2E,4E)-Methyl 5-((3,4-Dimethyl-5-Oxo-2,5-Dihydrofuran-2-Yl)Oxy)-4-Methylpenta-2,4-Dienoate
BOPIDY	4,4-difluoro-4-bora-3α,4α-diaza-s-indacene
HR	BF2 Chelate of (Z)-5-(3,5-dimethyl-1Hpyrrol-2-yl)-N-(4-((E)-1,4-dimethyl-2-((4-methyl-5-oxo-2,5-dihydrofuran-2-yloxy)methylene)-3-oxo-1,2,3,4-tetrahydrocyclopenta[b]indol-7-yl)phenyl)-5-(3,5-dimethyl-2H-pyrrol-2-ylidene)pentanamide
EG	BF2 Chelate of (E)-6-((3,5-dimethyl-1H-pyrrol-2-yl) (3,5-dimethyl-2H-pyrrol-2-ylidene)methyl)-2,2-dimethyl-2H-pyran-4(3H)-one

*Both the common name and the IUPAC name of each compound are listed. When an abbreviation for the compound is available, it is included in the parenthesis following the corresponding common name*.

In consistence with being a selective agonist, pyrabactin is an activator for only a subset of PYR/PYL ABA receptors (Park et al., 2009; Melcher et al., 2010). Moreover, it is intriguing that while pyrabactin is an agonist of PYR1 and PYL1, it is an antagonist of PYL2, competitively blocking ABA-dependent PYL2 activation (Melcher et al., 2010). This unique property of pyrabactin was exploited by Melcher et al. (2010) to decipher the mechanism of ABA receptor antagonism at the molecular level by the combinatorial approaches of structural, biochemical and molecular biological studies. They elaborately showed that it is the closed or open conformation adopted by the ligand-bound receptor that determines activation or inhibition of the ABA receptor. This antagonism model is complementary to the perception-activation mechanism of ABA receptors revealed by ABA *per se*, providing a full view of the mechanisms underlying receptor perception and activity regulation. Furthermore, based on this rational model of ABA receptor agonism and antagonism, virtual screening and docking analysis followed by *in vitro* validation has identified at least four pyrabactin-based small molecules as novel ABA-receptor agonists (compounds #32, #68, #71, and #98 in Figure 1A; Melcher et al., 2010), highlighting the efficacy of the application of pyrabactin as a chemical tool in ABA biology.

The same small molecule however, named differently as quinabactin or ABA MIMIC 1 (AM1), was identified as a new synthetic selective ABA agonist in two independent chemical library screens where a yeast two-hybrid assay and an *in vitro* protein interaction assay was applied, respectively (Figure 1A; Cao et al., 2013; Okamoto et al., 2013). This compound possesses broader receptor spectrum activity and increased bioactivity relative to pyrabactin, although both quinabactin/AM1 and pyrabactin belong to the sulfonamide type of compounds (Cao et al., 2013; Okamoto et al., 2013). On one hand, unlike pyrabactin's unique selectivity on seed germination in *Arabidopsis*, the physiological effects of quinabactin/AM1 are highly similar to those of ABA, triggering substantial ABA-like responses in vegetative tissues and promoting drought tolerance in adult plants (Cao et al., 2013; Okamoto et al., 2013). Based on their ligand-free oligomeric states, cytosolic ABA receptors can be divided into two major classes: PYR1 and PYL1-PYL3 are homodimers in solution, whereas PYL4-PYL12 are monomers (Miyakawa et al., 2013). Biochemical and genetic analyses showed that quinabactin's ABA-mimic effects in vegetative tissues are primarily mediated by dimeric ABA receptors (Okamoto et al., 2013). Thus, the use of quinabactin/AM1 as a selective agonist for a restricted subset of ABA receptors, i.e., dimeric ABA receptors, facilitates the revelation of the critical role of dimeric receptors in mediating ABA responses in vegetative tissues. On the other hand, although both quinabactin/AM1 and pyrabactin are sulfonamides, their chemical structures differ from one another: the naphthalene double ring and pyridine ring at each end of the sulfonamide linker in pyrabactin are replaced by a dihydro-quinolinone ring and benzyl group, respectively, in quinabactin/AM1 (Figure 1A). Comparison between the crystal structures of quinabactin/AM1- and pyrabactin-receptor-PP2C ternary complexes revealed that the binding mode of quinabactin/AM1 with the receptor more closely mimics that of ABA than pyrabactin, which is consistent with their physiological effects. The binding features of similarities to ABA and differences to pyrabactin provide a structural basis for designing the next generation of ABA-selective agonists, which are potential chemical reagents applicable in drought stress management for agricultural crops (Cao et al., 2013). Very recently, a panel of ABA analogs, each with a bulky group substitution on a specific position around the ABA ring, was assembled as agonists with varying efficacy to probe the specific activities of PYR1/PYL receptor-PP2C complex pairs and the resultant physiological effects in *Arabidopsis* based on biochemical and physiological assays (Benson et al., 2014). The findings from this study provide a comprehensive view of ABA structure-activity and ABA receptor-physiological relationships, as well as modification principles for the future design of selective ABA agonists.

ABA antagonists are potential chemical tools not only for studying ABA perception and signal transduction, but also for resolving the roles of ABA in phytohormone cross-talk responses. In an early study, a stereoisomeric acetylenic analog of ABA, (-)-4(Z)-(4*S*,5*R*)-4-hydroxy-4-(5-hydroxy-3-methylpent-3-en-1-ynyl)-3,3,5-trimethylcyclohexanone (PBI-51; Figure 1A), was recognized to act as an ABA antagonist inhibiting ABA-regulated gene expression in cress seed germination (Wilen et al., 1993). This compound is useful for studying the relationship between osmotic stress and ABA in the regulation of seed development. In another chemical library screen designed to identify candidate chemicals capable of antagonizing ABA-induced gene expression, a small molecule [5-(3,4-dichlorophenyl)furan-2-yl]-piperidine-1-ylmethanethione (DFPM; Figure 1A) was identified. DFPM was characterized as a selective ABA antagonist for a subset of ABA responses, including ABA-responsive gene expression and ABA-regulated stomatal movement, by disrupting partial ABA signaling network (Kim et al., 2011). Further analyses established that the antagonistic effects of DFPM on ABA signal transduction are mediated through activation of the early plant immune system. These data suggest the existence of a crosstalk between biotic and abiotic stress signaling pathways, where activation of early components in plant innate immune pathways negatively regulates ABA-mediated abiotic stress responses. Therefore, the potent small molecule DFPM can be used as a chemical tool for mechanistic dissection of both plant immunity and ABA signaling interference (Kim et al., 2011). In fact, evidences provided by biochemical and electrophysiological analyses of DFPM inhibitory activity indicated that DFPM disruption of ABA signaling occurs at the level of or downstream of intracellular Ca^2+^ signaling (Kim et al., 2011).

Very recently, based on the well-characterized structural features of ABA receptor system, a new type of ABA analogs, i.e., 3′-alkylsulfanyl-substituted ABAs called AS*n* compounds with *n* representing the alkyl chain length, was created by the structure-guided rational design strategy (Takeuchi et al., 2014). Among them, 3′-hexylsulfanyl-ABA (AS6; Figure 1A) was clarified as a potent ABA antagonist. Except for the six-carbon alkyl chain, it is structurally nearly identical to ABA. This chemical characteristic makes AS6 bind to PYL in a highly similar way as ABA with a comparable affinity, while positions its long *S*-hexyl chain protruding out onto PLY's PP2C-interaction surface, preventing ABA-induced PYL-PP2C interaction, consequently blocking plant ABA responses (Takeuchi et al., 2014). In addition to the potential agrichemical value in regulating stress responses and seed germination for crops, AS6 provides a new tool for dissecting ABA's multiple roles, particularly in non-model systems lacking genetic resources.

### SA agonists and antagonists

SA (Figure 1B) is a phenolic phytohormone known for its primary function as an endogenous signal mediating plant defense responses against pathogens, as well as influencing responses to abiotic stresses and other important aspects of plant growth and development (Vlot et al., 2009; Rivas-San Vicente and Plasencia, 2011). A complex SA-mediated disease resistance signaling network has been identified in recent years, in which NON-EXPRESSOR OF PATHOGENESIS-RELATED GENES 1 (NPR1), a transcription co-regulator, plays a central role (Vlot et al., 2009; Pajerowska-Mukhtar et al., 2013). Intriguingly, both NPR1 and its paralogs NPR3 and NPR4, two adaptors that bridge between the CULLIN 3 (CUL3) ubiquitin E3 ligase and its substrate, function as the long-sought-for SA receptors (Fu et al., 2012; Wu et al., 2012; for review: Pajerowska-Mukhtar et al., 2013), while NPR1 protein levels are precisely controlled via CUL3^NPR3^- and CUL3^NPR4^-mediated turnover through the proteasome (Spoel et al., 2009). However, the detailed mechanisms of SA perception by distinct receptors under specific physiological conditions and the immediate downstream NPR1 regulation are still elusive.

A number of compounds have been developed as synthetic analogs of SA and employed in disease control for crop protection. Among them, 2,6-dichloroisonicotinic acid (INA; Figure 1B) and benzo-(1,2,3)-thiadiazole-7-carbothioic acid *S*-methyl ester (benzothiadiazole or BTH; Figure 1B) are two notable molecules that have also been widely used in studies interrogating components in SA signaling and response (Uknes et al., 1992; Lawton et al., 1996). Meanwhile, selective agonists have been proven as powerful tools to delineate the function of individual members of functionally redundant receptors. Using a high-throughput chemical screening strategy targeting selective identification of immune-priming compounds, Noutoshi et al. (2012) isolated imprimatinC chemicals, including two structurally similar molecules imprimatin C1 and C2 (Figure 1B), as partial agonists of SA. These compounds effectively induce the expression of SA-responsive defense-related genes and increase disease resistance in *Arabidopsis*, while exhibiting no effects on the positive feedback loops in SA signaling and antagonism to jasmonic acid (JA) signaling (Noutoshi et al., 2012). It has been known that elucidation of SA-mediated early defense signaling events is often hampered by various feedback loops and cross-talk with other phytohormones that modulate the SA signal (Vlot et al., 2009). Thus, imprimatinC compounds can potentially assist to better understand the molecular events involved in SA defense signaling. Furthermore, structure-activity relationship (SAR) analyses implicated that the potential downstream metabolites of imprimatinC compounds, including 4-chlorobenzoic acid (4-CBA), 3,4-dichlorobenzoic acid (3,4-DCBA) and their derivative 3,5-DCBA (Figure 1B), also act as partial agonists of SA with various potencies (Noutoshi et al., 2012). Therefore, imprimatinC compounds and their potential functional metabolites can serve as valuable tools to address the complexity intrinsic to the activities of SA receptors, providing insights into the mechanisms governing early SA perception and NPR1 regulation and its role in plant immune signaling.

### Auxin agonists and antagonists

**Figure 2:**
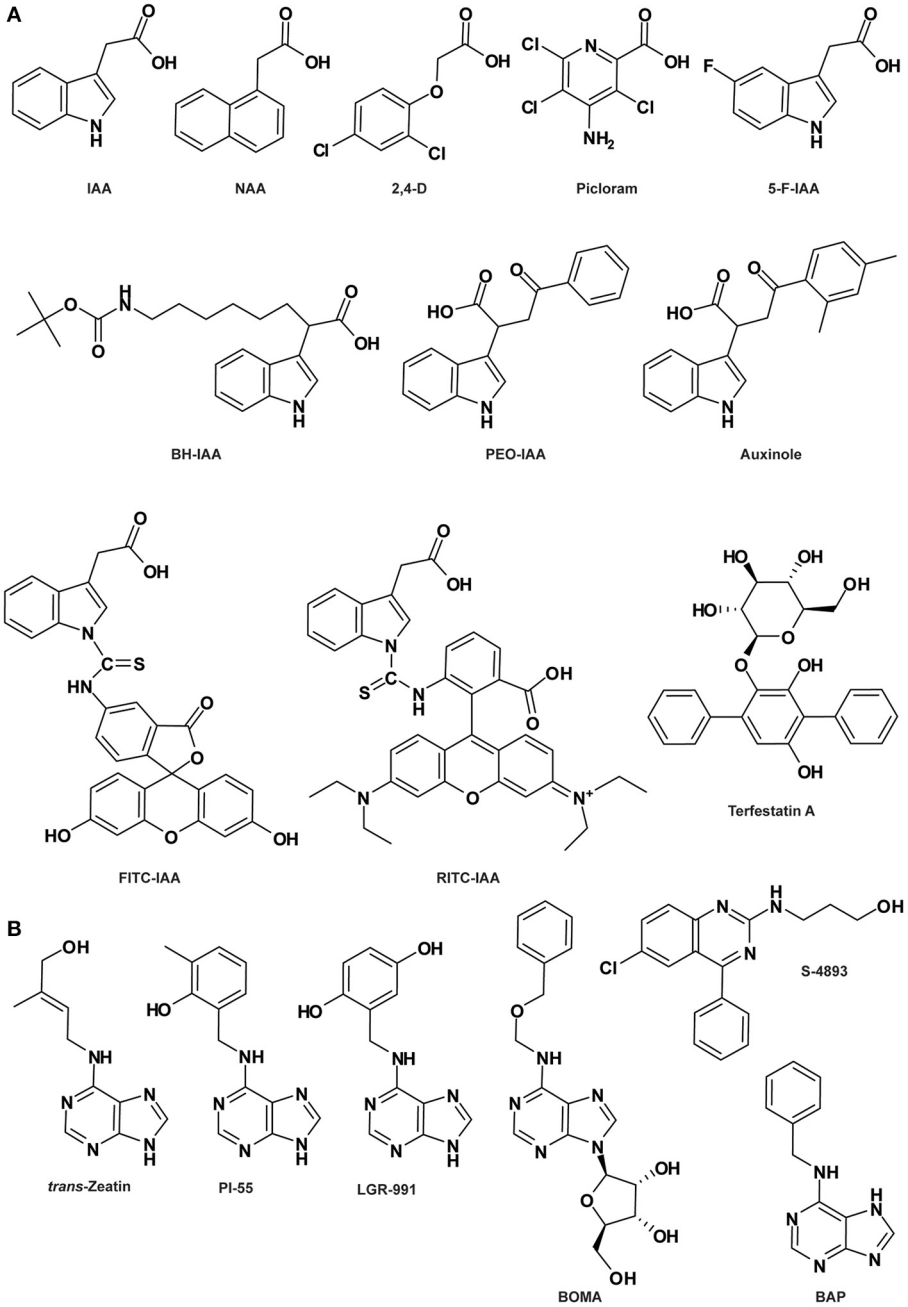
**Auxin- (A) and cytokinin-related compounds (B)**. See Table 1 for the full name of each compound.

Auxin is an important small-molecule phytohormone regulating almost every aspects of plant growth and development (Woodward and Bartel, 2005; Vanneste and Friml, 2009). Indole-3-acetic acid (IAA; Figure 2A) is the predominant form of naturally occurring auxin in plants, although indole-3-butyric acid (IBA), 4-chloroindole-3-acetic acid (4-Cl-IAA) and phenylacetic acid (PAA) have also been identified endogenously in different plant species (Simon and Petrášek, 2011). Elucidation of the cellular and physiological roles of auxin and its mode of action is historically reliant on the use of diverse bioactive small molecules, ranging from natural metabolites from plants or microbes to synthetic compounds. In recent years, the rapid development of chemical biology has contributed significantly to enhance our understanding of auxin biology, which has been comprehensively summarized in several recent reviews (De Rybel et al., 2009a; Hayashi and Overvoorde, 2013; Ma and Robert, 2014). Here, we intend to concentrate on the employment of auxin agonists and antagonists in interrogating the molecular mechanisms underlying auxin signaling and its regulation.

Auxin transcriptional response starts with the perception of the auxin ligand by the members of the auxin receptor protein family TRANSPORT INHIBITOR RESPONSE1 (TIR1)/AUXIN SIGNALING F-BOX1 (AFB1) to AFB5, which are F-box subunits of the S-PHASE KINASE-ASSOCIATED PROTEIN1-CULLIN1-F-BOX (SCF) type E3 ubiquitin ligase complex (Dharmasiri et al., 2005a,b; Kepinski and Leyser, 2005). This binding stabilizes the interaction between SCF^TIR1/AFB^ and co-receptors named AUXIN/INDOLE-3-ACETIC ACID INDUCIBLE (Aux/IAA) repressor proteins, which are negative regulators of auxin signaling (Abel et al., 1995; Gray et al., 2001; Tan et al., 2007). The ubiquitylation and subsequent degradation of Aux/IAA repressors via SCF^TIR1/AFB^-mediated 26S proteolysis removes the repression of (derepresses) activities of AUXIN RESPONSE FACTOR (ARF) transcription factors, leading to the transcription of downstream genes (Weijers et al., 2005; Szemenyei et al., 2008; Dos Santos Maraschin et al., 2009; Bargmann and Estelle, 2014). In this model, auxin behaves like molecular glue between the TIR1/AFB binding pocket and the recognition domain (DII) in the Aux/IAA proteins by stabilizing the co-receptor complex (Tan et al., 2007).

Various synthetic compounds capable of eliciting auxin-like responses were identified in the early years of auxin research and used as auxin agonists to examine and manipulate auxin signaling pathways (De Rybel et al., 2009a; Hayashi and Overvoorde, 2013; Ma and Robert, 2014), most notably 1-naphthaleneacetic acid (1-NAA) and the widely used herbicides 2,4-dichlorophenoxyacetic acid (2,4-D) and 4-amino-3,5,6-trichloro-2-pyridinecarboxylic acid (picloram) (Figure 2A). Genetic analyses of resistance to these compounds or their derivatives assisted in the isolation of a number of key components in auxin signaling, such as AUXIN-RESISTANT1 (AXR1) to AXR3, AXR5, AXR6, AFB4, and AFB5 (Estelle and Somerville, 1987; Woodward and Bartel, 2005). The highly selective resistance of either *afb4* and *afb5* to picolinate-type or *tir1* and *afb5* to benzoic acid-type synthetic auxins indicated that members of the auxin receptor family have different recognition specificities toward diverse auxinic molecules (Walsh et al., 2006; Gleason et al., 2011; Greenham et al., 2011). This was further corroborated by heterologous experiments using a yeast system showing that distinct auxin agonists differentially stabilize the TIR1-Aux/IAA co-receptor complex and AFB5 exhibits higher affinity to the synthetic auxin picloram (Calderón-Villalobos et al., 2012). Furthermore, based on auxin-dependent yeast 2-hybrid assays, biochemical properties of TIR1/AFB-Aux/IAA co-receptor complexes were systematically assessed, indicating that different co-receptor pairs yield a wide range of auxin-binding affinities which seem to be mainly governed by the Aux/IAA (Calderón-Villalobos et al., 2012). In *Arabidopsis*, there are 6 TIR1/AFBs and 29 Aux/IAAs; the cellular context-specific combinations between them may generate many co-receptors with distinct auxin-sensing capacities, resulting in distinct physiological effects (Bargmann and Estelle, 2014). Thus, agonists selectively affecting auxin-related physiological processes of interest represent novel chemical tools for examining specific aspects of auxin signaling.

The molecular structure and mechanism of auxin perception revealed by the crystallographic analysis of the auxin-bound co-receptor complex lay a good foundation for rational structure-based molecular design of auxin antagonists or anti-auxins, specifically blocking SCF^TIR1/AFB^-Aux/IAA-mediated nuclear auxin signaling. Three anti-auxins were generated by this strategy, i.e., *tert*-butoxycarbonylaminohexyl-IAA (BH-IAA), α-(phenylethyl-2-oxo)-IAA (PEO-IAA) and α-(2,4-dimethylphenylethyl-2-oxo)-IAA (auxinole) (Figure 2A), listed in order of increasing potency (Hayashi et al., 2008a, 2012b). These molecules bind with auxin receptors the same way as IAA, but prevent Aux/IAA docking and the formation of functional co-receptor complexes due to the hindrance caused by the alpha-substituted bulky groups. Thus, the competitive binding between anti-auxin and endogenous IAA inactivates the TIR1/AFB signaling pathway (Hayashi et al., 2008a, 2012b).

In parallel to the nuclear auxin receptors, the extracellular and cell surface-localized AUXIN BINDING PROTEIN1 (ABP1) has been proposed as another important receptor sensing extracellular auxin and mediating rapid non-transcriptional auxin responses centering on the plasma membrane (Sauer and Kleine-Vehn, 2011; Scherer, 2011), including auxin-induced inhibition of clathrin-mediated endocytosis (Robert et al., 2010) and auxin-dependent activation of RHO-RELATED PROTEIN OF PLANTS (ROP) Rho-GTPases governing cell polarity (Xu et al., 2010). It has also been shown that ABP1-mediated auxin signaling negatively regulates the SCF^TIR1/AFB^ pathway (Tromas et al., 2013). ABP1 was first purified from maize coleoptiles by immunoaffinity chromatography nearly 30 years ago (Löbler and Klämbt, 1985) and subsequently proven to bind auxin using photoaffinity labeling method (Jones and Venis, 1989). The crystal structure of ABP1 in complex with auxin was also resolved by Woo et al. (2002). Despite of these significant progresses, the molecular mechanism of ABP1-mediated auxin perception and signal transduction is mostly unresolved. Very recently, a breakthrough has been made on the characterization of the transmembrane kinase (TMK) receptor-like kinases as one group of the long-sought-after ABP1 docking proteins transmitting the extracellular ABP1-perceived auxin signal across the plasma membrane to induce cytoplasmic responses (Xu et al., 2014). Auxin binding to ABP1 prompts its interaction with the extracellular domain of TMK, forming an ABP1-TMK auxin perception complex on the cell surface that activates ROP activity and downstream signaling pathways (Xu et al., 2014). This groundbreaking finding opens a door for addressing many of the mysteries around this longest known but less characterized auxin signaling pathway.

It is reasonable to envision that chemical probes (agonists and antagonists) specifically targeting the ABP1 pathway, similar to those exemplified above for the SCF^TIR1/AFB^-Aux/IAA pathway, could enable identification of novel components in this pathway, shedding more light on ABP1-regulated aspects of auxin biology. In fact, two of such chemical probes have already been identified. *DR5* is a synthetic auxin-responsive element widely used to monitor nuclear TIR1/AFB-mediated auxin signaling (Ulmasov et al., 1997), while inhibition of clathrin-dependent PIN-FORMED (PIN) endocytosis is a hallmark phenomenon for ABP1-mediated auxin signaling. PEO-IAA is a specific antagonist of TIR1/AFB and therefore unable to induce *DR5* expression, but intriguingly inhibits clathrin-dependent PIN endocytosis (Robert et al., 2010), implying that it works as an agonist for ABP1. Conversely, 5-fluoroindole-3-acetic acid (5-F-IAA; Figure 2A), a halogenated IAA with auxin activity, is inactive in inhibiting PIN endocytosis while very effective in inducing *DR5* expression (Robert et al., 2010; Simon et al., 2013), functioning as an agonist for TIR1/AFB. Thus, the unique behaviors of these two bioactive molecules structurally analogous to IAA can be utilized in future studies to discriminate between nuclear TIR1/AFB- and extracellular ABP1-dependent auxin signaling pathways. Elucidation of the crystal structure of the auxin-bound ABP1-TMK perception complex will facilitate the development of ABP1-targeted auxin agonists and antagonists, representing novel tools for better understanding of the molecular events controlling this cell surface-cytoplasmic auxin perception and signaling system. Although the transmembrane feature of the TMK protein might impose some difficulties for protein crystallization, the finding that auxin-prompted physical interaction occurs between ABP1 and the extracellular domain of TMK could alleviate this problem to some extent (Xu et al., 2014).

### CK agonists and antagonists

CK are classical plant hormones responsible for the regulation of various aspects of plant growth and development such as cell division coordination, cell proliferation, seed germination and root and leaf differentiation (Mok and Mok, 2001; Werner et al., 2001, 2003). Based on the structure of the side-chain, natural CKs are adenine derivatives classified as isoprenoid or aromatic CKs. The isoprenoid CKs, such as *trans*-zeatin (tz; Figure 2B), are the ones most frequently found in plants. N^6^-benzyladenine (BA; also named 6-benzylaminopurine [BAP]; Figure 2B) and its derivatives, such as *meta*- and *ortho*-topolin and the most characterized CK kinetin, belong to the aromatic CKs (Sakakibara, 2006; Bajguz and Piotrowska, 2009; Lomin et al., 2012). Some derivatives of urea also display CK activity, like diphenurea and thidiazuron (Arata et al., 2010). CK signaling occurs through a phosphorylation cascade, which is initiated by the CK receptor HISTIDINE KINASE (HK). In Arabidopsis, three types of CK receptors have been identified: CYTOKININ RESPONSE 1 (CRE1), also called ARABIDOPSIS HISTINE KINASE 4 (AHK4), AHK2 and AHK3.

In the seventies, several synthetic CK derivatives, such as pyrazolo[4,3-d]pyrimidines (Hecht et al., 1971; Skoog et al., 1973), pyrrolo[2,3-d]pyrimidines (Iwamura et al., 1974, 1975) and 7-deaza analogs of 2-methylthioadenine CK (Skoog et al., 1975) were classified as anti-CKs. It was later shown that these compounds do not act as CK antagonists on CK receptors, as was initially suspected, but that at least some of them act as cyclin-dependent kinase inhibitors (Sṕichal et al., 2007; Arata et al., 2010). Recently, two BAP derivatives displaying anti-CK activity have been described (Sṕichal et al., 2009; Nisler et al., 2010). Among them, N^6^-(2-hydroxy-3-methylbenzylamino) purine (PI-55; Figure 2B) blocks the binding of the natural tz to the receptor CRE1/AHK4 in a competitive manner. PI-55 is also effective on root growth and branching and stimulates seed germination, supporting the notion that PI-55 inhibits CK perception *in planta*. Moreover, the antagonistic activity of PI-55 was also demonstrated in other species such as tobacco and wheat (Sṕichal et al., 2009). Despite its antagonistic effect on CRE1/AHK4, PI-55 at high concentration may weakly induce its interaction with AHK3, leading to AHK3 partial activation (Sṕichal et al., 2009). In contrast, another synthetic compound N^6^-(2,5-dihydroxybenzylamino) purine (LGR-991; Figure 2B), structurally similar to PI-55, acts as an antagonist to the CK receptor CRE1/AHK4 with the same efficiency as PI-55, while competitively antagonizing AHK3 (Nisler et al., 2010). In comparison, LGR-991 presents a lower agonistic effect on the expression of the *ARR5:GUS* reporter gene and consistently induces a phenotype related to a reduction of CK level/signaling. More recently, a synthetic analog of N^6^-adenosine, N^6^-(benzyloxymethyl) adenosine (BOMA; Figure 2B), was described as a novel anti-CK. BOMA is highly specific to CRE1/AHK4 but not AHK3, similarly to PI-55 (Krivosheev et al., 2012).

Interestingly, the phenylquinazoline derivative S-4893 (Figure 2B) has been characterized as a novel type of CK antagonist targeting the CK receptor CRE1/AHK4 in a non-competitive way (Arata et al., 2010). S-4893 has been suggested to bind to CRE1/AHK4 differently from the natural CK, and may prevent the conformational modifications of the CK receptor that are required to induce CK-mediated signal transduction. At the physiological level, S-4893 inhibits CK effects on root growth and callus formation in Arabidopsis and other species such as rice (Arata et al., 2010).

Over the past few years, the discovery of synthetic molecules modulating CK signaling has considerably increased our knowledge of CK perception and provided new opportunities to better understand CK biology.

### BR agonists and antagonists

**Figure 3:**
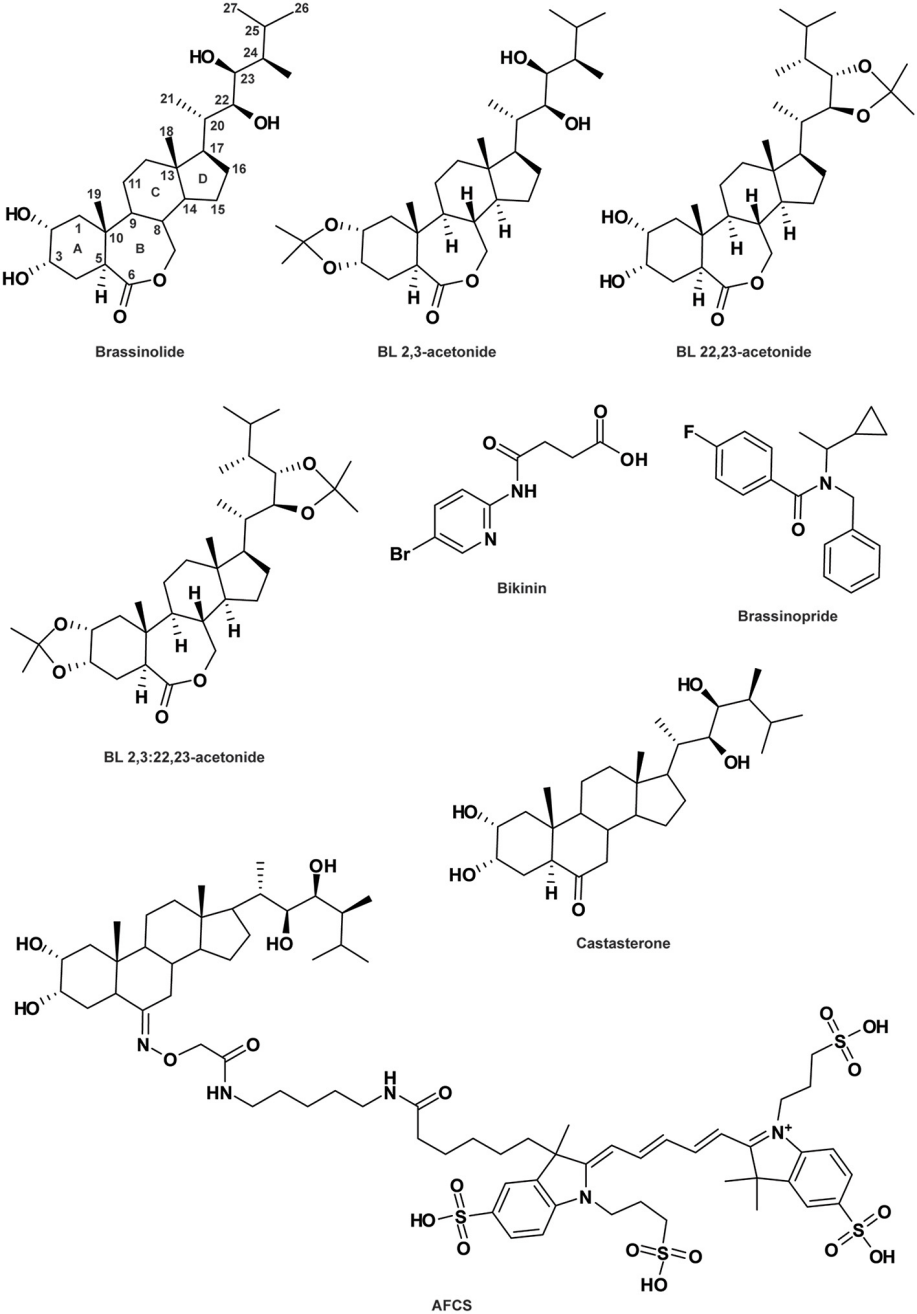
**Brassinosteroid-related compounds**. See Table 1 for the full name of each compound.

BRs are steroid plant hormones that regulate cell division, elongation and differentiation and are essential for development of organs such as the shoot/hypocotyl, root, leaf and pollen tube. Additionally, BRs are involved in developmental and environmental responses like senescence and biotic and abiotic stress integration (Yang et al., 2011). BRs are perceived by the extracellular domain of the receptor BRASSINOSTEROID INSENSITIVE 1 (BRI1), leading to its dissociation from and association with BRI1 KINASE INHIBITOR 1 (BKI1) and BRI1-ASSOCIATED RECEPTOR KINASE 1 (BAK1; also named SOMATIC EMBRYOGENESIS RECEPTOR KINASE 3 [SERK3]), respectively. Phosphorylation of BRI1 is required for the complete activation of the BR signaling pathway (Yang et al., 2011; Wang et al., 2012). Chemical screens based on *Arabidopsis* hypocotyl elongation identified modulators of BR response such as the activator bikinin (BIK; Figure 3; De Rybel et al., 2009b) and the inhibitor brassinopride (BRP; Figure 3; Gendron et al., 2008). BIK triggers BR signaling by binding to the adenosine tri-phosphate (ATP) pocket of the major BR-signaling regulator BR-INSENSITIVE2 (BIN2), thus preventing phosphorylation of the downstream transcription factor BRI1-EMS-SUPPRESSOR1 (BES1; De Rybel et al., 2009b). However, BRP's mode of action remains elusive.

Among all the endogenous BRs, brassinolide (BL; Figure 3) is the most potent. However, a decrease in its bioactivity can be induced by the engineered modifications of 2-O, 3-O, 22-O or 23-O-methylation (Back et al., 2002; Back and Pharis, 2003). Crystal structure analysis of the BRI1-BL complex revealed that the reduction in the activities of these structural analogs might be due either to their inhibitory effects on the BAK1/SERK3-BRI1 interaction or their lower affinity for BRI1 itself (Hothorn et al., 2011; She et al., 2011; Muto and Todoroki, 2013). To distinguish between these two hypotheses, 2,3-acetonide-BL, 22,23-acetonide-BL and 2,3:22,23-acetonide-BL (diacetonide) were produced, all showing no BL-like activity (Figure 3; Muto and Todoroki, 2013). However, 2,3-acetonide-BL and to some extent, 22,23-acetonide-BL, display BL antagonist behavior. The potential activity of diacetonide could not be tested due to its high hydrophobic property preventing it from crossing the cell wall. The weaker antagonist activity of 22,23-acetonide-BL compared to 2,3-acetonide-BL strongly suggests that the 2,3-dihydroxyl group is central for its interaction with the receptor BRI1 (Muto and Todoroki, 2013). Furthermore, it was demonstrated that the interaction between BRI1 and SERK1 is promoted by the presence of BL, which acts as a molecular glue (Santiago et al., 2013). Within SERK1, the residue Phe^61^ and its closest histidine interact with the BL C-ring and the 2α,3α vicinal diol moiety of the hormone, respectively (Santiago et al., 2013). However, whether both hydroxyl groups at C-2 and C-3 or only one of them is required for a potent antagonist effect remains elusive. Taken together, these studies demonstrate the possibility to improve the understanding of BL signaling via chemically modulating BL-BRI1 interaction.

### SL agonists and antagonists

**Figure 4:**
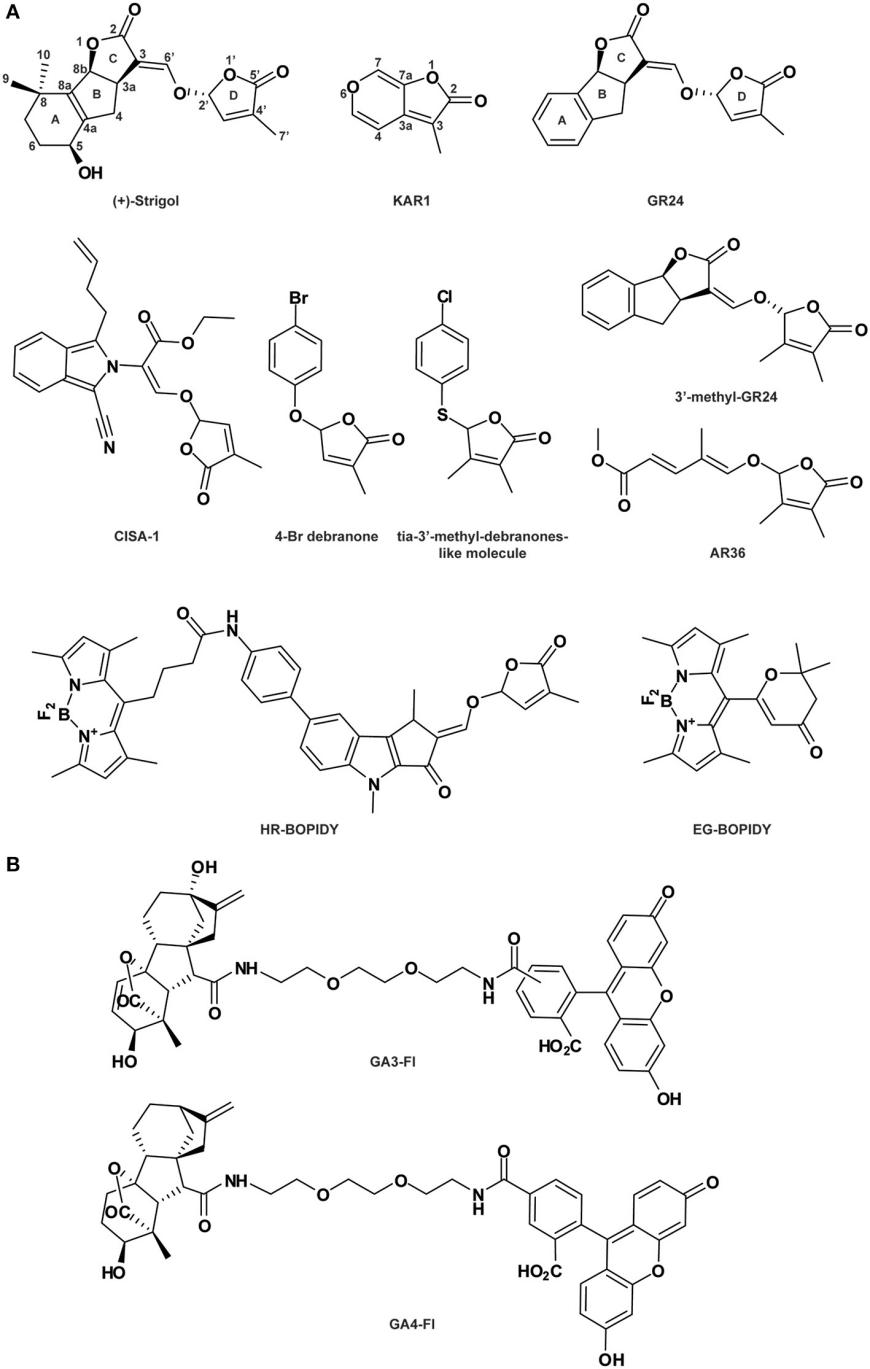
**Strigolactone- (A) and gibberellin-related compounds (B)**. See Table 1 for the full name of each compound.

The group of SL-related molecules has been described as being involved in general plant development such as root growth, stem secondary development and leaf senescence (Seto et al., 2012). Additionally, SLs act as signals in the rhizosphere for both parasitic and symbiotic interactions (Xie et al., 2010). Karrikins (KARs) and SLs are natural plant signaling molecules involved in common processes such as seed germination and seedling photomorphogenesis (Nelson et al., 2012; Seto et al., 2012; Waters et al., 2014). KARs have been identified in the smoke of burning vegetation and cannot be strictly considered as phytohormones. Both types of molecule contain an enol ether and a substituted methyl butenolide ring, both essential for their stimulatory activity on seed germination (Figure 4A). However, KAR structure is simpler than that of SL: the butenolide moiety of KAR is fused to a pyran ring, while it is connected to a tricyclic lactone (ABC-ring) in SL (Figure 4A). Although SL and KAR signaling processes are mediated by a common unique F-box protein MORE AXILLARY GROWTH 2 (MAX2), MAX2 is coupled with one of two distinct α/β-hydrolase fold proteins, depending on the phytohormone: DECREASED APICAL DOMINANCE 2 (DAD2)/DWARF14 (D14) for SL or KARRIKIN INSENSITIVE 2 (KAI2) for KAR (Nelson et al., 2011; Hamiaux et al., 2012; Waters et al., 2012). This particular example demonstrates that small structural differences within natural compound enable high specificity for receptor and co-receptor interaction.

As substantial quantities of natural SLs are difficult to obtain, SL synthetic analogs have been engineered. Among them, GR24, in which the A-ring is substituted by an aromatic ring (Figure 4A), is the main SL-like compound currently used. As does endogenous SL, GR24 interacts with and is cleaved by the α/β-hydrolase fold protein DAD2/D14 (Hamiaux et al., 2012; Kagiyama et al., 2013; Zhao et al., 2013). Cyano-isoindole-strigolactone-analog-1 (CISA-1) is structurally related to nijmegen-1 (Figure 4A; Nefkens et al., 1997) and has also been shown to act through a MAX2-mediated signaling pathway (Rasmussen et al., 2013). Remarkably, CISA-1 is more active and stable than GR24, and possesses interesting fluorescent properties (Rasmussen et al., 2013, see also the “Labeled molecules: compelling tools to understand the action of signaling molecules” section). Moreover, novel SL analogs have been identified as specifically targeting the plant developmental processes via a MAX2-dependent signaling pathway, such as 4-Br debranone (5-[4-bromophenoxy]-3-methylfuran-2[5H]-one) (Figure 4A; Fukui et al., 2011, 2013), 3′-methyl-GR24, tia-3′-methyl-debranones-like molecule and AR36 (Figure 4A; Boyer et al., 2012, 2014). Their weak potencies on rhizosphere define them as promising SL plant growth regulators (Fukui et al., 2013; Boyer et al., 2014).

## From structure to activity

SAR analyses investigate the relation between a molecule's structure and its bioactivity by testing the potency of multiple natural or synthetic analogs and have been widely used in medical chemistry, pharmacology, cosmetics, toxicology and environmental science (Hasdenteufel et al., 2012). Additionally, determination of the active moieties sheds light on their modes of action. By this means, analogs can be identified and purchased in open-access databases or designed and synthesized by combining chemistry.

### SAR to reveal the importance of the complexity

BRs are plant steroid hormones containing a 5α-cholestane carbon skeleton with a side chain at the C17 position. The first steroidal lactone, named BL (Figure 3), was isolated in 1979 from *Brassica napus* pollen (Grove et al., 1979) and since then, more than 50 natural BRs have been characterized throughout the plant kingdom (Fujioka, 1999; Bajguz and Tretyn, 2003). They present some natural variations in the side chain and the substituents in the A and B-rings (Figure 3). To better understand BR mode of action, structural requirements for BR bioactivity have been widely studied by the establishment of numerous bioassays including rice leaf lamina inclination and elongation, and curvature and splitting of the bean second internode (Mandava, 1988; Zullo and Adam, 2002; Back and Pharis, 2003). First of all, the trans-A/B-ring conformation, the presence and spatial position of the oxygen atom on the B-ring, and the importance of the 2α, 3α vicinal diol moiety on the A-ring have been shown to be essential for providing BL-like activity (Mandava, 1988; Baron et al., 1998; Seto et al., 1999; Back and Pharis, 2003; Bajguz, 2011). Regarding the B-ring structure, natural BRs are divided into four types including 7-oxalactone, 6-oxo (6-ketone), 6-deoxo (non-oxidized) and 6-hydroxy. Interestingly, the 6-oxo BRs (such as castasterone [CS]; Figure 3; Yokota et al., 1982) display a lower potency than the 7-oxalactone type (for example BL; Bajguz, 2011), suggesting that the seven-membered B-ring lactone is required for optimum activity. The non-oxidized BRs do not show any activity (Bajguz, 2011). Additionally, a substitution of the C-6-keto (in the B-ring) by an α or β hydroxyl group is not favorable for BR potency, indicating that the presence of an electronegative charge is required to maintain a high level of chemical activity (Ramirez et al., 2005). On the other hand, the side chain of the steroid nucleus is also involved in BR activity determination. Bioactivity is mostly maintained for the BR lacking the methyl group at the C26, C27, or C28, as well as the one presenting a methylidene- or ethylidene- substitution at C24 (Back and Pharis, 2003). Moreover, 2-O, 3-O, 22-O or 23-methylation critically reduces bioactivity (Back et al., 2002; Back and Pharis, 2003). Although the SAR analyses reveal possibilities to modulate BR structure, it appears that each part of the BR chemical structure is a major actor in bioactivity determination. Additionally, new insights into the molecule-receptor binding mechanisms revealed by crystal structure analyses support previous SAR analysis data (Hothorn et al., 2011; She et al., 2011).

### SAR to reveal specific activity

SAR analyses hold great potential for dissecting the functions of endogenous compounds. Several natural SLs have been identified throughout the plant kingdom with a large spectra of activities including the promotion of parasitic weed seed germination, arbuscular mycorrhizal (AM) fungus branching induction and plant growth regulation (Zwanenburg and Pospíšil, 2013). The common structure of endogenous SL includes a tricyclic lactone (ABC-ring) connected via an enol ether bridge to a butenolide group (the D-ring; see Strigol, Figure 4A). Importantly, it has been shown that structural requirements to specific activity are divergent.

The active core (or bioactiphore) of SL to stimulate germination of parasitic weeds such as *Orobranche* and *Striga* species has been determined by multiple SAR analyses (Zwanenburg et al., 2009; Janssen and Snowden, 2012; De Saint-Germain et al., 2013). Endogenous SL and the structurally simplified SLs, GR24 (replacement of the A-ring by an aromatic ring), GR7 (lacking the A-ring), GR5 (completed deletion of A and B-rings; Johnson et al., 1981), ABC scaffold and D-ring (2-ethyxybutenolide; Zwanenburg et al., 2009) have been analyzed. This SAR investigation has revealed that the CD but not the ABC part of the molecule is sufficient for seed germination stimulation, suggesting that the SL active core resides in the CD group (Mangnus and Zwanenburg, 1992; Mangnus et al., 1992; Zwanenburg et al., 2009; Zwanenburg and Pospíšil, 2013). The original SL D-ring must be preserved, as the C4 methyl group is essential for SL potency on seed germination (Mangnus and Zwanenburg, 1992; Zwanenburg et al., 1994).

SAR analysis was also conducted on SL to understand SL activity as a plant hormone controlling shoot branching (Fukui et al., 2011; Boyer et al., 2012, 2014). As for root parasitic seed stimulation, the D-ring is essential for shoot branching bioactivity and small changes in the C3 could affect interaction with the receptor. Surprisingly, presence of substitutions on the A and B-rings and a change in the stereochemistry on the C2 do not affect bioactivity (Boyer et al., 2012; Chen et al., 2013). According to Boyer et al. (2012), the SL structure could be replaced by the D-ring only for bud outgrowth inhibition. In agreement, SL analogs (Debranones) in which the D-ring is only linked to an aromatic cycle present the same bioactivity as GR24 for both rice and *Arabidopsis* branching inhibition (Fukui et al., 2011, 2013) and for pea shoot branching inhibition (Boyer et al., 2014). Additionally, the ABC-part could be substituted by an unsaturated acyclic carbon chain without affecting the shoot branching inhibition on pea (Boyer et al., 2014).

Remarkably, the SAR analysis results on AM fungus branching induction are slightly divergent from those mentioned above. As for SL-dependent germination stimulation, the D-ring is required (Akiyama et al., 2010) and the SL stereochemistry is critical (De Saint-Germain et al., 2013). Additionally, the enol-ether bridge connecting the C-D-ring is also critical for SL optimum function (Kondo et al., 2007; Akiyama et al., 2010). However, modifications of the ABC substructure (in particular the A-ring) drastically diminish bioactivity (Besserer et al., 2006; Akiyama et al., 2010; Prandi et al., 2011; Cohen et al., 2013; Boyer et al., 2014). As an example, GR5 stimulates *Orobranche* seed germination but does not induce hyphal branching in AM fungus assays (Johnson et al., 1976; Akiyama et al., 2010). Then, the structure requirement for AM branching is highly specific and small modifications induce a drastic effect on the bioactivity (Boyer et al., 2014).

Overall, the SAR studies performed on multiple endogenous and synthetic SLs reveal that the structural requirements, as an effector of plant development, AM fungal branching or root parasitic seed germination present some noticeable differences. Accordingly, news SL analogs mimicking specific SL activities could be synthetized such as done by Fukui and co-authors (Fukui et al., 2011, 2013). Overall these studies demonstrate that that SL signaling functions through distinct modes of perception in different systems (Boyer et al., 2012, 2014; Chen et al., 2013; Cohen et al., 2013; De Saint-Germain et al., 2013).

### SAR to uncouple hormonal crosstalks

SAR analysis can also disentangle crosstalk between hormone-mediated pathways. As an example, BRP has been characterized as not only a BR signaling inhibitor (see the “Agonist and antagonist molecules” section) but also an inducer of ethylene response (Gendron et al., 2008). Interestingly, one of the BRP derivatives targets essentially the ethylene signaling pathways, highlighting the potential of close structural analogs to separate diverse targeted pathways (Gendron et al., 2008).

The SAR is a powerful approach for dissecting the modes of action of signaling molecules. Indeed, SAR analysis results in the discovery of the required moiety for bioactivity. Interestingly, this approach could also lead to the identification of “dead analogs” (Toth and Van Der Hoorn, 2010). For example, the investigation of several pyrabactin derivatives revealed that its bioactivity requires the pyridyl nitrogen, as the apyrabactin analog (Figure 1A) is inactive (Park et al., 2009). Additionally, these totally inactive “dead analogs” could be essential controls in biological assays. In other cases, SAR analysis helps in the design of new antagonist derivatives such as 2,3-acetonide-BL (Muto and Todoroki, 2013) described earlier.

## Labeled molecules: compelling tools to understand the action of signaling molecules

The determination of the required structure for a molecule's bioactivity by SAR analysis is central for the successful design of active tagged/labeled compounds (see as an example in bacteria Chorell et al., 2012). In animal biology, fluorescence-labeled ligand analogs are currently used to study the localization of their receptors as well as the distribution of active endogenous molecules. For example, this strategy was used in research on the dopamine transporter (DAT) involved in dopamine re-uptake. The neurotransmitter DAT is also the principal target for psychostimulants such as cocaine (Chen et al., 2004; Gether et al., 2006; Torres and Amara, 2007). The conception of fluorescent cocaine analogs was essential to permit direct visualization of DAT and to directly follow its cellular trafficking, as no efficient antibody or labeled protein could be generated (Eriksen et al., 2009). The production of fluorescent analogs also creates possibilities to visualize the uptake and *in vivo* distribution of molecules, as illustrated by the use of a fluorescence-tagged glucose probe (Kim et al., 2012).

### Fluorescent labeled molecules

The synthesis of fluorescent or tagged compounds has become increasingly attractive for plant researchers over the past few years and has provided new tools to unravel phytohormone signaling and distribution. Several endeavors to generate fluorescent auxin conjugates have been successful. The first attempt was conducted by Muscolo and co-authors, who synthesized fluorescein isothiocynate (FITC) conjugates of IAA and humic substances potentially able to interact with auxin receptors (Muscolo et al., 2007). More recently, new fluorescent auxin conjugates have been produced by coupling with FITC (Figure 2A) or rhodamine isothiocynate (RITC; Figure 2A; Sokolowska et al., 2014). These two conjugates present an auxin-like activity and are transported via the auxin transport machinery, making them promising tools to study auxin transport and function *in planta*. IAA-FITC and IAA-RITC are both stable at room temperature, however the electrospray ionization tandem mass spectrometry (ESI-MS) analysis conducted on IAA-FITC revealed a degradation of the auxin conjugates. According to the authors, the reason for this may be that the ESI process itself directly reduces the stability of most conjugates. However, the potential IAA-FITC fragmentation *in planta* must be considered.

Since 2009, Bhattacharya and co-authors have generated a new class of bioactive SL analogs named PL series, some of which present luminescent properties under UV radiation at 360 nm (Bhattacharya et al., 2009; Prandi et al., 2011). These compounds are generated by substitution of various functional groups on the A and C-rings of the SL ABC nucleus and provide valuable data for SAR analysis. Although all these analogs show bioactivity as stimulators of germination in *Orobranche aegyptiaca* and hyphal branching in *Gigaspora margarita*, their luminescent properties are not suitable for observation using microscopy-based analysis. However, based on these results, other fluorescently labeled SL analogs have been designed and used successfully *in vivo* in plants and fungi (Prandi et al., 2013). Four new molecules have been produced using different fluorophores inserted on the aromatic ring, which include 5-dimethylaminophtalene-1-sulfyl (dansyl) for (E)-N-(4-(1,4-dimethyl-2-(((4-methyl-5-oxo-2,5-dihydrofuran-2-yl)oxy)methylene)-3-oxo-1,2,3,4-tetrahydrocyclopenta[b]indol-7-yl)phenyl)-5-(dimethylamino)naphthalene-1-sulfonamide (AO), the fluorophore fluorescein for (E)-5-(3-(4-(1,4-dimethyl-2-((4-methyl-5-oxo-2,5-dihydrofuran-2-yloxy)methylene)-3-oxo-1,2,3,4-tetrahydrocyclopenta[b]indol-7-yl)phenyl)thioureido)-2-(6-hydroxy-3-oxo-3H-xanthen-9-yl)benzoic acid(BL), and 4,4-difluoro-4-bora-3α,4α-diaza-s-indacene (BOPIDY) for the molecules BF2 Chelate of (Z)-5-(3,5-dimethyl-1Hpyrrol-2-yl)-N-(4-((E)-1,4-dimethyl-2-((4-methyl-5-oxo-2,5-dihydrofuran-2-yloxy)methylene)-3-oxo-1,2,3,4-tetrahydrocyclopenta[b]indol-7-yl)phenyl)-5-(3,5-dimethyl-2H-pyrrol-2-ylidene)pentanamide (HR) and BF2 Chelate of (E)-6-((3,5-dimethyl-1H-pyrrol-2-yl) (3,5-dimethyl-2H-pyrrol-2-ylidene)methyl)-2,2-dimethyl-2H-pyran-4(3H)-one (EG). The two tagged molecules HR-BOPIDY and EG-BOPIDY (Figure 4A) show strong stimulatory effects on *Phelipanche aegyptiaca* seed germination. Additionally, their absorption-emission spectra are suitable for confocal analysis. HR and EG are efficiently taken up by *Medicago truncatula* root hairs and show a cytoplasmic distribution. During the same time period, a new fluorescent SL named CISA-1 has been synthesized by a simple procedure (Figure 4A; Rasmussen et al., 2013). A classical genetic approach performed on *Arabidopsis* Columbia wild-type, *max1*/*max4* (SL-deficient mutants) and *max2* (SL-insensitive mutant) confirms its SL-like activity. Similarly to GR24, CISA-1 reduces the number of adventitious roots and inflorescence stems in the SL-deficient mutants, while the SL-insensitive mutant *max2* is not affected. These data suggest that CISA-1 acts downstream of MAX1 and MAX4 through a MAX2-dependent signaling pathway. Furthermore, like GR24, CISA-1 suppresses *MAX4* expression after 24 h of treatment, probably due to feedback regulation from the increased endogenous SL level (Umehara et al., 2008; Mashiguchi et al., 2009). The fluorescent property of CISA-1 has been observed at 10 mM in solution with the excitation and emission spectra between 300-380 nm and 400 nm, respectively, but unfortunately fluorescence detection *in planta* still needs to be improved (Rasmussen et al., 2013).

Two fluorescently labeled bioactive gibberellins (FLBG) have been synthesized with different spacers (1,4-dithiobutylene or 1,3-dithiopropylene chain) between the fluorescein and the gibberellin (GA) molecule (Pulici et al., 1996). Interestingly, the FLBG with the longer chain displayed a stronger GA activity, suggesting that the implementation of a long spacer facilitates the interaction between the active GA moiety and its receptor. Later on, this fluorescence-labeled GA was used tomonitor the potential cell-to-cell movement of GA and its role in releasing chilling-induced dormancy of *Betula pubescens* (Rinne et al., 2001). Very recently, two other bioactive and stable fluorescent GAs were generated (GA_3_-Fl and GA_4_-Fl; Figure 4B) and used to analyze the spatial distribution of GA in *Arabidopsis* roots (Shani et al., 2013). According to studies on the stability of the GA_3_ conjugates, the fluorescein has been linked via an amide bond to the GA_3_ molecule on the C6 position (Liebisch et al., 1988). The same strategy was also used for GA_4_. These two labeled compounds are bioactive due to the existence of an intact GA molecule within their structures, retaining their interaction with the GA receptor. However, they are not suitable substrates for *in vivo* GA metabolism, making them ideal to study GA transport processes. After application, labeled GAs accumulate in the endodermis layer within the elongation zone of the root (Shani et al., 2013). Pharmacological studies combined with the analysis of mutants defective in endodermal cell layer identity revealed that the GA accumulation is regulated by an active mechanism (Shani et al., 2013). Furthermore, by using fluorescent GAs, it was confirmed that GA distribution is regulated by ethylene, adding another dimension to GA function in plant development (Shani et al., 2013). This study elegantly demonstrates how fluorescently labeled GAs can help to dissect GA localization and real time transport *in planta*.

Recently, a bioactive fluorescently labeled BR analog named Alexa Fluor 647-castasterone (AFCS; Figure 3) has been produced to analyze BR signaling processes (Irani et al., 2012). The position of the fluorophore AF467 at the C6 of the B-ring of CS was chosen based on previously generated biotin-tagged photoaffinity CS and is in accordance with the ligand-binding pocket structure of the receptor BRI1 (Kinoshita et al., 2005; Hothorn et al., 2011; She et al., 2011). AFCS was validated as a bioactive BR, although its potency is lower than that of the native BR or CS. AFCS internalization has been shown to be mediated by BRI1, as its uptake is increased in plants overexpressing the BR receptor and reduced in the *bri1* mutant. This fluorescently tagged BR thereby enabled visualization of the ligand-receptor interaction via AFCS-BRI1. In addition, it revealed internalization of the BR-BRI1 complex by live imaging, which is dependent on clathrin-mediated endocytosis and ADP-ribosylation factor-guanine nucleotide exchange factors (ARF-GEFs) (Irani et al., 2012). This study validates the potential of fluorescently labeled compounds not only to dissect hormone transport, but also to visualize ligand-receptor interaction *per se*, as well as trafficking of the ligand-receptor complex.

Labeled molecules are valuable tools to identify direct targets of bioactive endogenous or synthetic compounds. In particular, the application of biotin-tagged compounds facilitates the isolation of compound targets such as receptors by affinity chromatography and could even lead to the determination of the molecule-binding site.

### Tagged molecules

Reizelman et al. (2003) have produced a plethora of tagged SLs with radioactive, photoaffinity, biotin and fluorescent (dansyl) groups to isolate the SL receptor. Germination assays on *Striga hermonthica* seeds revealed that bioactivity of the labeled analogs is retained, demonstrating that the SL binding site tolerates a large substituent on the SL A-ring. Although a 60 kDa membrane-bound protein was isolated by the authors as a SL receptor in *Striga hermonthica* seeds (Zwanenburg et al., 2009; Zwanenburg and Pospíšil, 2013), direct evidence is not yet available and further experiments are required to confirm these results. Nevertheless, the synthesis and use of biotin-tagged photoaffinity CS (BPCS) has helped to demonstrate the direct binding between BRI1 and physiologically active BRs (Kinoshita et al., 2005). BPCS is a bioactive CS analog containing a carbene-generating phenyldiazirine moiety and a biotin tag, which allows its detection by an anti-biotin antibody. Under UV radiation, the phenyldiazirine moiety enables covalent liaison between BPCS and the binding region of the specific receptor. Binding analyses using BPCS, ^3^H-labeled BL and recombinant BRI1 fragments were performed to characterize the minimum required region for BR perception. These data showed that the minimum region required is composed of 94 amino acids in the extracellular domain of BRI1 constituted by the island domain (70 amino acids located between the 21st and 22nd leucine-rich repeat [LRR] domain of BRI1) and LRR 22. However, structural analysis of the steroid complex demonstrates that the hormone-binding site is larger than this initial prediction (Hothorn et al., 2011).

Interestingly, not only phytohormone analogs but also compounds with antagonist activity such as Terfestatin A (TrfA; Figure 2A) can be used to isolate cognate receptors (Yamazoe et al., 2004, 2005). TrfA has been shown to disturb auxin signaling independently from the canonical auxin receptor TIR1 (Yamazoe et al., 2005). Therefore, it can be exploited to identify novel auxin receptors. Determination of the active core of TrfA by SAR analysis could provide the possibility to design a biotin-tagged active TrfA or a solid support-linked TrfA suitable for affinity chromatography of the direct target protein (Hayashi et al., 2008b). Nevertheless, no results using this tagged compound have yet been published.

### Caged molecules

Recently, development of novel technologies based on the creation of caged compounds has created the possibility to control the distribution of active compounds in a temporally and spatially (at the intracellular level) defined way. Caged compounds display an inducible activity as a result of the photo-removable structure, which blocks their functional groups but is easily released by photolysis. This is very useful for modulating the intracellular level of a molecule within a single cell and for investigating the direct consequences of these changes at the cellular level. The design of the cage is a critical step, as the caged compound must be soluble, cell permeable and stable. Diverse bioactive elements have been caged and extensively used, such as messenger ribonucleic acid (mRNA), deoxyribonucleic acid (DNA), nucleotides, peptides, calcium, neurotransmitters and inositol (Ellis-Davies, 2007). Over the past few years, the synthesis of caged auxin, GA, ABA, JA, and SA have been described (Ward and Beale, 1995; Allan et al., 1998). However, detailed biological properties are not provided for all of them. The bioactivity of caged ABA has been successfully validated in stomata guard cells (Allan et al., 1994, 1998). More recently, novel caged auxin (Kusaka et al., 2009) and caged CK (Hayashi et al., 2012a) have been engineered and their bioactivity has been verified by bioassays using specific hormone-responsive marker *Arabidopsis* lines. The caged hormones could be used as a trigger to control hormonal distribution inside the cell, making them potential tools to detail the hormone's cellular response. These caged molecules could thereby help to gain a better comprehension of hormone function, adding new strategies to dissect hormone-mediated signaling.

Taken together, these studies demonstrate that labeled/tagged hormone analogs can be helpful toward a better understanding of hormone biology, in particular with respect to hormone signaling and transport mechanisms. Indeed, fluorescent analogs enable a direct visualization of the tempo-spatial distribution and/or intracellular trafficking of the ligand-receptor complex. However, some fluorescently labeled analogs require further structural modifications to achieve the spectrometric properties suitable for live imaging studies. The development of new dyes with enhanced characteristics should be explored to generate new conjugates with stronger signal and sensitivity. Modified growth regulators carrying a biotin tag would also be helpful for isolating the direct target protein by affinity chromatography and for determining the binding site of the known receptor. Furthermore, the use of these compounds overcomes several laboratory problems, such as the difficulty to obtain efficient antibodies against the receptor, the long time needed to produce transgenic lines with tagged receptors and the safety issues related to radio-labeled molecules.

## Conclusions

**Figure 5:**
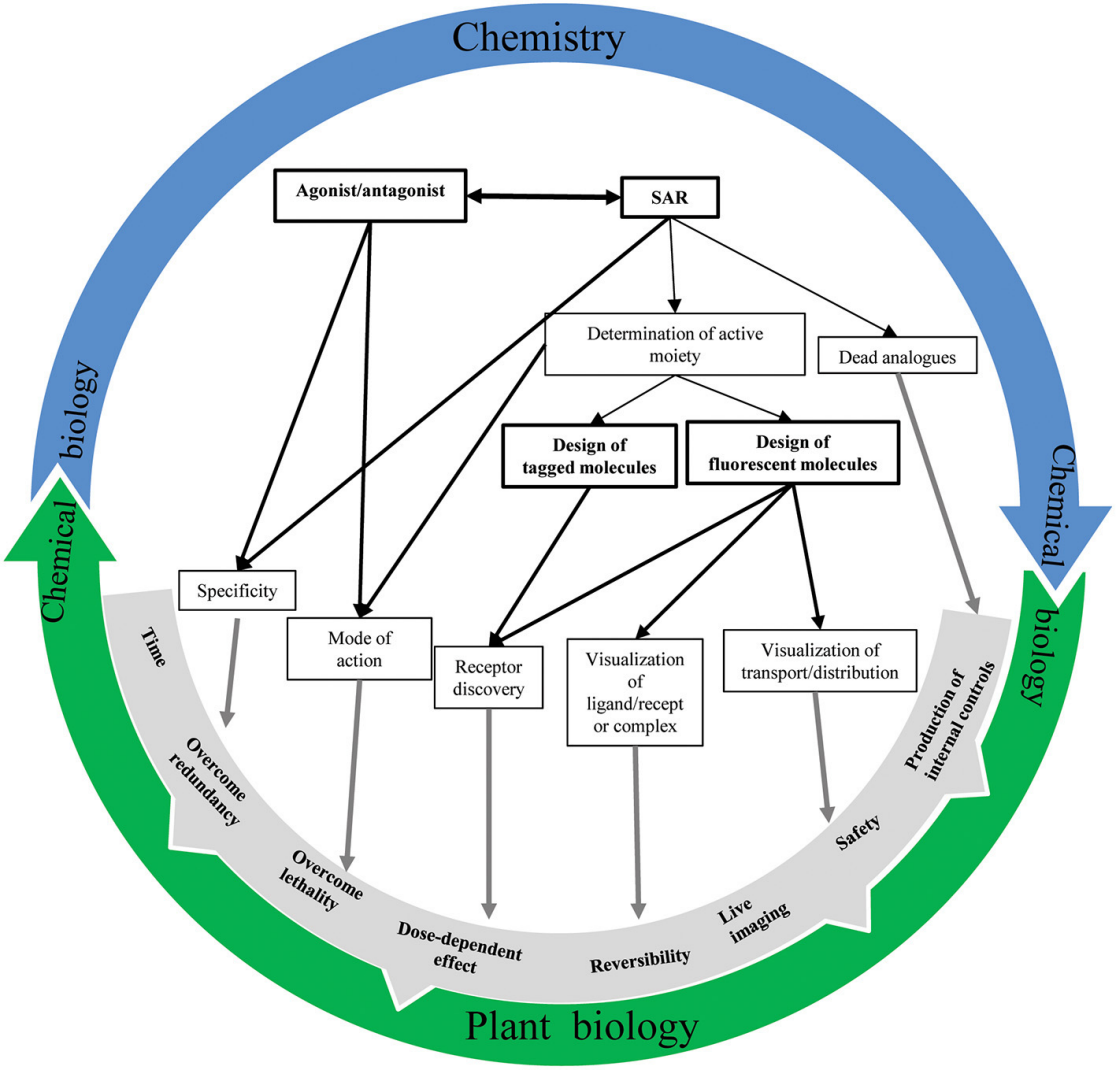
**Chemistry-plant biology relationship**. An overview of the interconnection possibilities between chemistry and biology to better understand phytohormone signaling mechanisms.

Our understanding of plant hormone signaling has been advanced tremendously by the use of small molecules (Figure 5). Increased knowledge of phytohormone structure has provided essential information such as the hormone's chemical properties and its active moiety. Ultimately, these details combined with structural characterization of the target protein facilitate the rational design of new derivatives targeting one specific component of the signaling pathways. Additionally, the engineering of labeled analogs can enable the isolation of hormone receptors and the direct visualization/monitoring of the hormone's tempo-spatial distribution as well as the ligand-receptor complex localization. Remarkably, subtle changes in plant hormone structure count and promote the possibility to precisely dissect the hormone's signaling pathways and the discovery of new endogenous actors. However, it should be noted that structural changes of a molecule could affect tremendously its binding affinity to the receptor, its transport or diffusion rate as well as the way it is uptaken and modified by the metabolic machinery. Along with the expansion of metabolomic technologies and a full coverage of endogenous molecule space, chemical biology will become essential for a better understanding of the molecular mechanisms governing phytohormone regulation. Computerized modeling of potential receptor structure in association with *in silico* molecule docking analysis has opened the door for the systematical investigation of hormone-mediated signaling pathways in plants. In this way, a tight collaboration between chemistry and plant biology is vital toward enhancing our understanding of plant hormone signaling.

### Conflict of interest statement

The authors declare that the research was conducted in the absence of any commercial or financial relationships that could be construed as a potential conflict of interest.
